# A Teen With Trisomy 18: Challenges and Triumphs of a Long Life With Edwards Syndrome

**DOI:** 10.7759/cureus.77417

**Published:** 2025-01-14

**Authors:** Wayne B Tate, Kaylie Ward, Zairha G Snider

**Affiliations:** 1 Pediatrics, Edward Via College of Osteopathic Medicine, Blacksburg, USA; 2 Pediatrics, Carilion Clinic, Radford , USA

**Keywords:** congenital heart defects, edwards syndrome, genetics, intractable seizures, karyotype, perinatal, trisomy 18

## Abstract

Trisomy 18, also known as Edwards syndrome, is a rare genetic disorder resulting from an extra copy of chromosome 18. It is associated with severe congenital malformations, distinct clinical features, and high morbidity and mortality. Despite the odds, we describe the case of a 16-year-old female patient with trisomy 18. During a well-child visit with her pediatrician, the patient's parents express concerns about worsening seizure activity. Her seizures began at the age of nine and were initially well managed with anti-epileptics but eventually progressed to refractory generalized and myoclonic epilepsy, resulting in the placement of a vagus nerve stimulator (VNS) at the age of 13. Though this intervention initially extended seizure-free periods, they have gradually increased in frequency and duration since its placement. Additional past medical and surgical history is pertinent for Dandy-Walker malformation, omphalocele repair, corrected bowel malrotation, gastrostomy tube dependence, genitourinary malformations with recurrent urinary tract infections (UTIs), a bicuspid aortic valve, and surgical correction of three congenital heart defects: an atrial septal defect (ASD), a ventricular septal defect (VSD), and patent ductus arteriosus (PDA). Little is known about factors associated with long-term survival in patients with trisomy 18, emphasizing the importance of cases like ours that highlight the evolving clinical course, the importance of trisomy 18-specific growth curves, complex medical and surgical management, and efforts to support and encourage caregivers as these patients age.

## Introduction

Trisomy 18 (Edwards syndrome) is a genetic disorder caused by an abnormal number of chromosomes and is associated with numerous congenital anomalies and developmental disorders that are a constant threat to life. Very few affected individuals survive their first year of life. Aneuploidy can be further classified as complete (94%), mosaic (<5%), or partial trisomy (2%), which may vary in disease severity. Duplication of the long arm (18q) of the chromosome is commonly thought to cause disease, as Edward syndrome arising from an extra copy of the short arm (18p) has yet to be described [1]. Second only to trisomy 21 (Down syndrome), which occurs in roughly one out of 700 live births, trisomy 18 occurs in roughly one out of 7,000 live births [2].

Phenotypically, the syndrome is largely characterized by a prominent occiput, severe neurologic impairment, clenched hands, micrognathia, structural heart defects, and rocker-bottom feet. Management of patients with Edwards syndrome is often demanding on caregivers and requires a well-coordinated medical and surgical effort across disciplines. Death most commonly results from a combination of cardiac failure secondary to congenital malformations and respiratory insufficiency from central apnea or airway obstruction [3]. With historically poor prognoses and multiple comorbidities, aggressive surgical management has been frequently debated with a low threshold for palliative care involvement. Though advancements in prenatal screening have increased prevalence, less than 10% of newborns survive their first year, with very few reports of patients over the age of five [4]. We describe a case of a 16-year-old with trisomy 18 who has far outlasted her life expectancy.

## Case presentation

A 16-year-old female with complete trisomy 18 confirmed by karyotype presented to her pediatrician for a well-child check. She is nonverbal and fully dependent on her parents for assistance with activities of daily living. Her parents mentioned that they had become concerned about the rising frequency and duration of her seizures despite close management by pediatric neurology for refractory myoclonic and generalized epilepsy. A vagus nerve stimulator (VNS) was implanted three years ago and allowed for a significant reduction in seizure activity, though her condition had gradually worsened since the procedure. Although her seizures began at age nine, our patient continually progressed in development and functional status, including nearly walking on her own, until the age of 11 when her parents noted an apparent encephalopathic event that triggered a regression of neurologic development, worsening seizures, and a plateau in physical development. Given her condition, palliative care had been consulted prior to her visit to ensure their daughter's comfort and maximize her quality of life. Their recommendations of adding lorazepam for breakthrough seizures and avoiding nighttime feedings were discussed. During her visit, she was scheduled for a follow-up electroencephalogram (EEG) the following week, which revealed an overall increase in epileptogenicity and breakthrough seizures despite a regimen of rufinamide, clobazam, cannabidiol, and a functioning VNS. Given previous failed trials of levetiracetam and topiramate and an adverse reaction to lamotrigine, her pediatric neurologist subsequently increased her clobazam dosage and discussed deep brain stimulation, which the family tentatively declined.

Past medical history was notable for severe physical and intellectual limitations, Dandy-Walker malformation, Lennox-Gastaut syndrome, neurogenic bladder, recurrent respiratory and urinary tract infections, obstructive sleep apnea, and hearing loss despite cholesteatoma excision. Shortly after birth, she underwent an omphalocele repair, correction of bowel malrotation, and gastrostomy tube placement, on which she is still dependent. Other surgical interventions included an appendectomy, cholecystectomy, resection of a renal abscess with hydronephrosis, liver wedge biopsy for elevated transaminases, and bladder stone removal. Cardiovascular anomalies included a bicuspid aortic valve, atrial septal defect (ASD), ventricular septal defect (VSD), and patent ductus arteriosus (PDA). Although her family was initially counseled to prepare for death at birth and not to pursue surgical intervention, her ASD, VSD, and PDA were eventually repaired at three months of age.

In the clinic, she was seated calmly in her father’s arms and remained cheerful. Her vitals at this visit were a blood pressure of 98/62 mmHg, heart rate of 112 beats per minute, respiratory rate of 18 breaths per minute, and weight of 39 pounds. Physical examination revealed a thin, nonverbal female with bilateral spastic paralysis, a prominent occiput, micrognathia, bilateral amblyopia with retained visual acuity, and low-set ears with mild hearing loss. Further examination revealed clear breath sounds bilaterally and no murmurs, rubs, or gallops on auscultation of her heart. Inspection of her extremities revealed clenched hands, flexion contractures of her knees, club feet, and a positive Galeazzi sign, denoting right-sided hip dysplasia for which she was followed by orthopedics and performed physical therapy. Her stature and weight by age were plotted using the Centers for Disease Control and Prevention (CDC) standardized female (two to 20 years) growth chart and were reviewed during the visit. At all points, she was noted to remain below the third percentile for stature and the first percentile for weight, both plateauing at 11 years of age. 

## Discussion

Trisomy 18 is a rare genetic syndrome caused by trisomy of the 18^th^ chromosome. Up to 90% of cases present with recognizable craniofacial and musculoskeletal abnormalities such as clenched hands, rocker-bottom feet, a prominent occiput, and low-set ears. However, congenital cardiac anomalies, cerebral malformations, and disorders within the gastrointestinal and genitourinary systems are often present and greatly increase morbidity and mortality [5]. Such disorders demand life-long commitment from caregivers and often require frequent visits to various specialists. Though there have been reports of long-term survival with trisomy 18, the various factors that result in some individuals living longer than others have yet to be elucidated.

**Figure 1:**
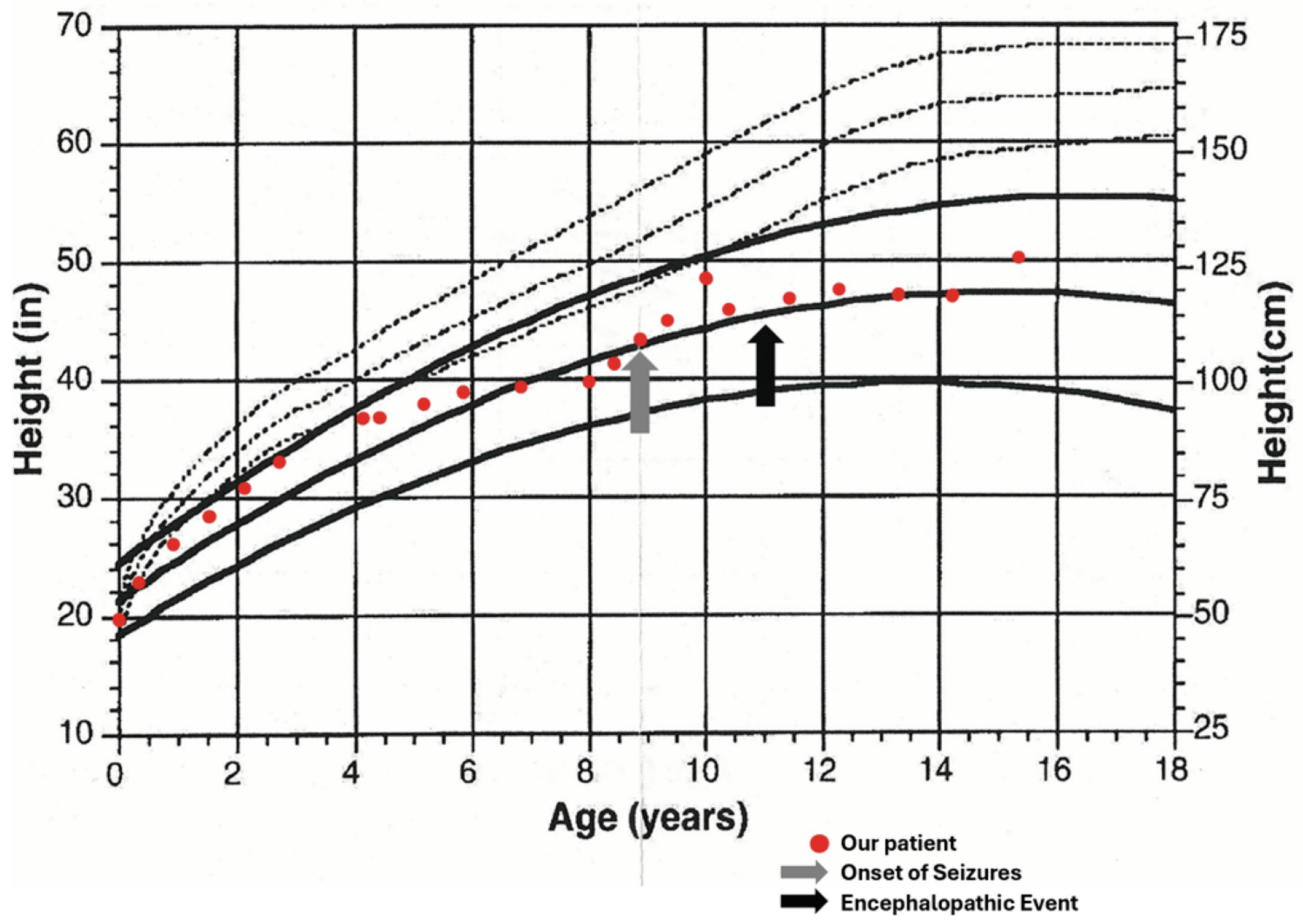
Trisomy 18-specific growth curve denoting height-for-age This figure depicts the plotted height (inches) for age (years) of a 16-year-old female patient with trisomy 18 on a trisomy 18-specific growth curve [6]. Red dots represent her individual height data collected at the corresponding time points. The gray arrow denotes the onset of her seizures, whose gradual worsening is the reason for her visit. The black arrow denotes an unknown insult to her brain resulting in a stark regression in development. Solid lines denote an expected regression with 95% confidence intervals for those with trisomy 18, while dotted lines represent that of an unaffected child. Source [6]; Reproduced with permission of John Wiley & Sons, Inc.

**Figure 2:**
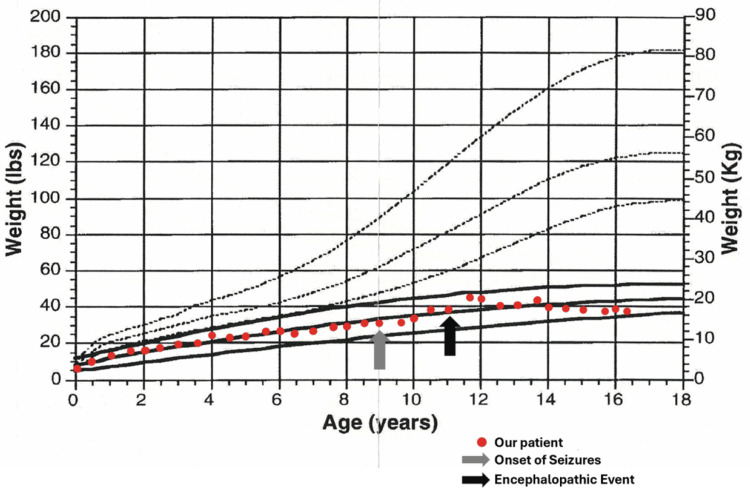
Trisomy 18-specific growth curve denoting weight-for-age This figure depicts the plotted weight (kg) for age (years) of a 16-year-old female patient with trisomy 18 on a trisomy 18-specific growth [6]. Red dots represent her individual weight data collected at the corresponding time points. The gray arrow denotes the onset of her seizures, whose gradual worsening is the reason for her visit. The black arrow denotes an unknown insult to her brain resulting in a stark regression in development. Solid lines denote an expected regression with 95% confidence intervals for those with trisomy 18, while dotted lines represent that of an unaffected child. Source [6]; Reproduced with permission of John Wiley & Sons, Inc.

Children with trisomy 18 have lower birth weights than unaffected children and develop more slowly. Growth curves specific to trisomy 18 may provide valuable insight into her growth relative to her peers [6]. Despite having appeared severely developmentally impaired when plotted on a standard CDC growth curve, her height (Figure 1) and weight (Figure 2) progressed normally when compared to others with trisomy 18. This emphasizes the importance of trisomy 18-specific assessments to more accurately monitor growth and development. 

Surgical management is beginning to overtake a predominantly palliative approach at birth that was previously recommended based on anticipated short life expectancy [7]. Though offering cardiac surgery to trisomy 18 patients has been historically controversial, most centers now offer surgical intervention to even the most complex of cardiac abnormalities in trisomy 18 patients, many of which are finding improved outcomes [8]. Despite being initially counseled to pursue palliative care upon her birth, her family quickly opted for surgical management after lengthy discussions with various specialists in the area, correcting her bowel malrotation and omphalocele in her first week of life; ASD and VSD repair with PDA ligation, gastrostomy tube placement, and cholecystectomy with liver biopsy all within four months of life; and VNS placement for refractory seizures at the age of 13. While these interventions may have contributed to such unusual longevity, the impact of a loving and supportive family cannot be overstated.

Requiring lifelong support and specialized care, chronic illness takes a physical, emotional, and financial toll on every family, particularly the caregivers [9]. Though our patient’s family is no exception, they actively pursue the support of others and ways to share their experience. They are involved with the Support Organization for Trisomy (S.O.F.T.) and have created opportunities to advocate and educate at the medical school level regarding trisomy 18, informed decision-making, and the many lessons their daughter has taught them.

Positively associated with maternal age, Edwards syndrome has gradually increased in prevalence as the average maternal age has increased globally [10, 11]. Additional risk factors include a previous pregnancy affected by trisomy 18 and a family history of chromosomal translocations [3]. Interestingly, as overall prevalence has increased, live birth prevalence has decreased, a dichotomy likely resulting from advancements in prenatal detection, intrauterine demise, and elective termination of pregnancies [12]. A 2021 study by Fick and Sexton noted a decrease in mortality and an overall increase in procedures undergone by trisomy 18 patients over the last 20 years. However, it remains unclear if such factors are related [13]. As these patients live longer, reports such as this denoting medical and surgical management amongst the evolving complexities and comorbidities associated with long-term survival may inform medical practice, growth and developmental assessments, advocacy efforts, and education.

## Conclusions

Trisomy 18 is a rare genetic syndrome characterized by three copies of chromosome 18, with distinct clinical features and a high mortality rate. With less than 10% surviving their first year of life and very few documented cases of survival beyond five years, the report of our 16-year-old patient surviving well beyond her life expectancy may aid in prognostic counseling and clinical decision-making while reinforcing the need for standardized assessment protocols. Though further research is needed to better understand the factors promoting long-term survival, cases like ours provide valuable insights into the dynamic disease course, long-term sequelae, and management as patients with Edwards syndrome age. 
